# Interobserver agreement between artificial intelligence models in the thyroid imaging and reporting data system (TIRADS) assessment of thyroid nodules

**DOI:** 10.1007/s12020-025-04272-1

**Published:** 2025-05-15

**Authors:** Andrea Leoncini, Pierpaolo Trimboli

**Affiliations:** 1 Clinic for Radiology, Imaging Institute of Southern Switzerland, Ente Ospedaliero Cantonale (EOC), Bellinzona, Switzerland; 2 Thyroid Unit, Clinic for Endocrinology and Diabetology, Ente Ospedaliero Cantonale (EOC), Bellinzona, Switzerland; 3 Faculty of Biomedical Sciences, Università della Svizzera Italiana, Lugano, Switzerland

**Keywords:** Thyroid, Ultrasound, TIRADS, Artificial Intelligence, Inter-observer agreement

## Abstract

**Background:**

As ultrasound (US) is the most accurate tool for assessing the thyroid nodule (TN) risk of malignancy (RoM), international societies have published various Thyroid Imaging and Reporting Data Systems (TIRADSs). With the recent advent of artificial intelligence (AI), clinicians and researchers should ask themselves how AI could interpret the terminology of the TIRADSs and whether or not AIs agree in the risk assessment of TNs. The study aim was to analyze the interobserver agreement (IOA) between AIs in assessing the RoM of TNs across various TIRADSs categories using a cases series created combining TIRADSs descriptors.

**Methods:**

ChatGPT, Google Gemini, and Claude were compared. ACR-TIRADS, EU-TIRADS, and K-TIRADS, were employed to evaluate the AI assessment. Multiple written scenarios for the three TIRADS were created, the cases were evaluated by the three AIs, and their assessments were analyzed and compared. The IOA was estimated by comparing the kappa (κ) values.

**Results:**

Ninety scenarios were created. With ACR-TIRADS the IOA analysis gave κ = 0.58 between ChatGPT and Gemini, 0.53 between ChatGPT and Claude, and 0.90 between Gemini and Claude. With EU-TIRADS it was observed κ value = 0.73 between ChatGPT and Gemini, 0.62 between ChatGPT and Claude, and 0.72 between Gemini and Claude. With K-TIRADS it was found κ = 0.88 between ChatGPT and Gemini, 0.70 between ChatGPT and Claude, and 0.61 between Gemini and Claude.

**Conclusion:**

This study found that there were non-negligible variability between the three AIs. Clinicians and patients should be aware of these new findings.

## Introduction

Thyroid nodules (TNs) occur commonly, and ultrasound (US) evaluation is the most accurate tool for assessing their risk of malignancy (RoM) [1]. The reliability of US has been largely proven, so international societies have published various US-based classification systems, which are generally reported through use of the Thyroid Imaging and Reporting Data System (TIRADS) terminology [2–4], with the intent to 1) standardize the lexicon for US reporting and 2) improve the selection of TNs for fine-needle aspiration cytology (FNAC). Currently, an important project concerns the creation of a universal lexicon (phase I) and, later, an international TIRADS (I-TIRADS) [5].

With the recent advent of artificial intelligence (AI) systems [6], clinicians and researchers should ask themselves how AI could interpret the terminology of a TIRADS and whether or not AIs agree in the risk assessment of TNs. It is also of interest to know if AIs differ in their assessment, which involves interpretation of TIRADS terms. Indeed, several online sites and many applications based on TIRADS lexicon have been diffused, and both clinicians and patients increasingly use those tools. Given that the three major TIRADSs were conceived for use in different contexts and have been associated with different behaviors [7], significant differences in AI interpretation of TIRADS terms is quite important. Studies have investigated the interobserver variability between medical operators who rated US images or clips of TNs. Solymosi et al. found Cohen κ value ranging between 0.26 and 0.71 [8], Persichetti et al. found a Cohen κ value of 0.42 for the ACR-TIRADS and of 0.39 for the EU-TIRADS [9], and Grani et al. found Cohen κ value of 0.61, 0.68, and 0.82 when using ACR-TIRADS, EU-TIRADS, and K-TIRADS, respectively, for FNAC [10]. However, no studies have systematically evaluated the interobserver agreement (IOA) between AIs in assessing the RoM of TNs across various TIRADSs categories according to their written US descriptions. Indeed, since operators and patients can use AI to assess the TN RoM and then make a decision according to the AI result, obtaining reliable information about the IOA between AIs has become essential.

The study aim was to analyze the IOA between three AIs in the assessment of TNs across the categories of the most diffused TIRADSs.

## Methods

### Study design

Three AIs were compared: ChatGPT, Google Gemini, and Claude, which are advanced AI models developed by leading technology companies for natural language processing. ChatGPT, developed by OpenAI, is based on the GPT architecture and uses deep learning and transformer models to generate human-like text. Gemini, developed by Google DeepMind, combines large language models with reinforcement learning techniques for more adaptive interactions. Claude, created by Anthropic, emphasizes AI alignment and safety through constitutional AI principles. These systems are trained on vast datasets and use supervised and unsupervised learning. The systems generate context-aware responses by predicting text sequences and leveraging attention mechanisms. Their applications span medicine, research, and communication.

The three most well-known TIRADSs, ACR-TIRADS [2], EU-TIRADS [3], and K-TIRADS [4], were compared to evaluate the possible differences in AI performance. As the study aim was to evaluate performance of and IOA between three AIs, the case series was no real but created combining the descriptors of the three TIRADSs. The study design included a three-step procedure: 1) multiple scenarios for each of the three TIRADS were created by randomly combining their US descriptors; 2) the cases were evaluated by the three AIs, each using the three TIRADSs to assess them; 3) the AI assessments were analyzed and compared, and their IOA was calculated.

### Ethics

This study did not include data from humans, so the approval of ethics committees was waived.

### Statistical analysis

The IOA was estimated by kappa (κ) values: κ = 0, no agreement; κ = 0–0.20 none to slight; 0.21–0.40: fair; κ = 0.41–0.60 moderate; κ = 0.61–0.80 substantial; κ = 0.81–1.0 almost perfect. The sample size was calculated for an expected κ of 0.40, with a confidence interval of 95% [11]; accordingly, a minimum sample of 21 cases was sufficient to evaluate each TIRADS. The 2023 version of MedCalc (MedCalc Software Ltd., Belgium) was used to perform the statistical analysis.

## Results

**Fig. 1:**
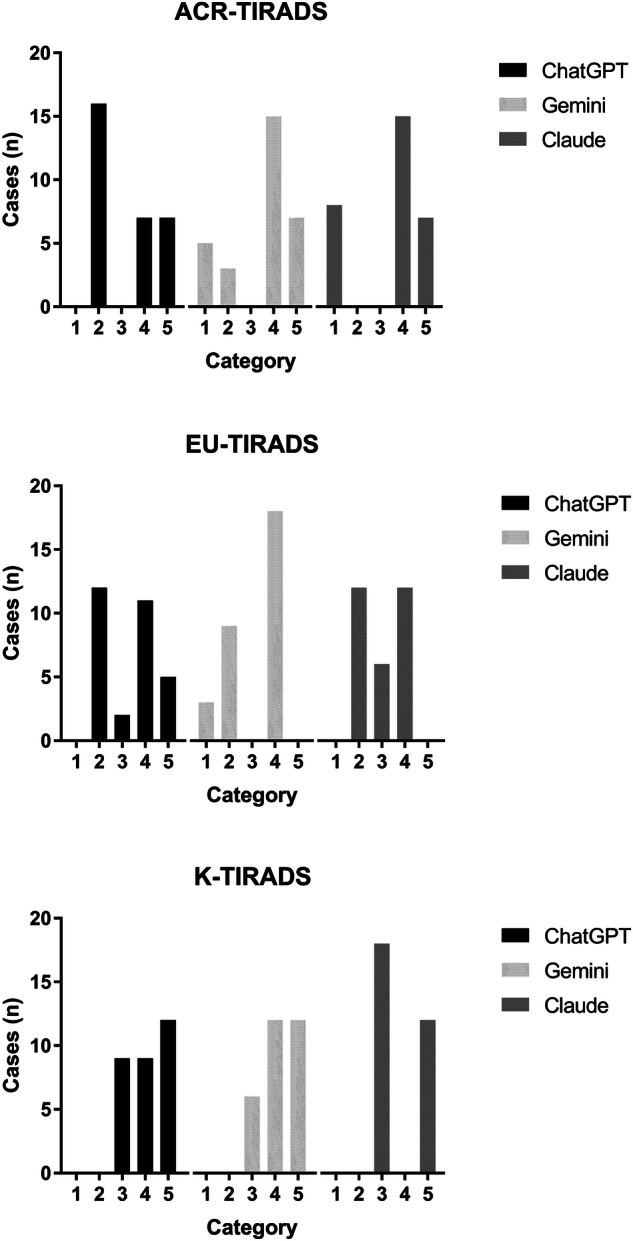
Distribution of thyroid nodules according to AIs assessment across the TIRADSs categories

**Fig. 2:**
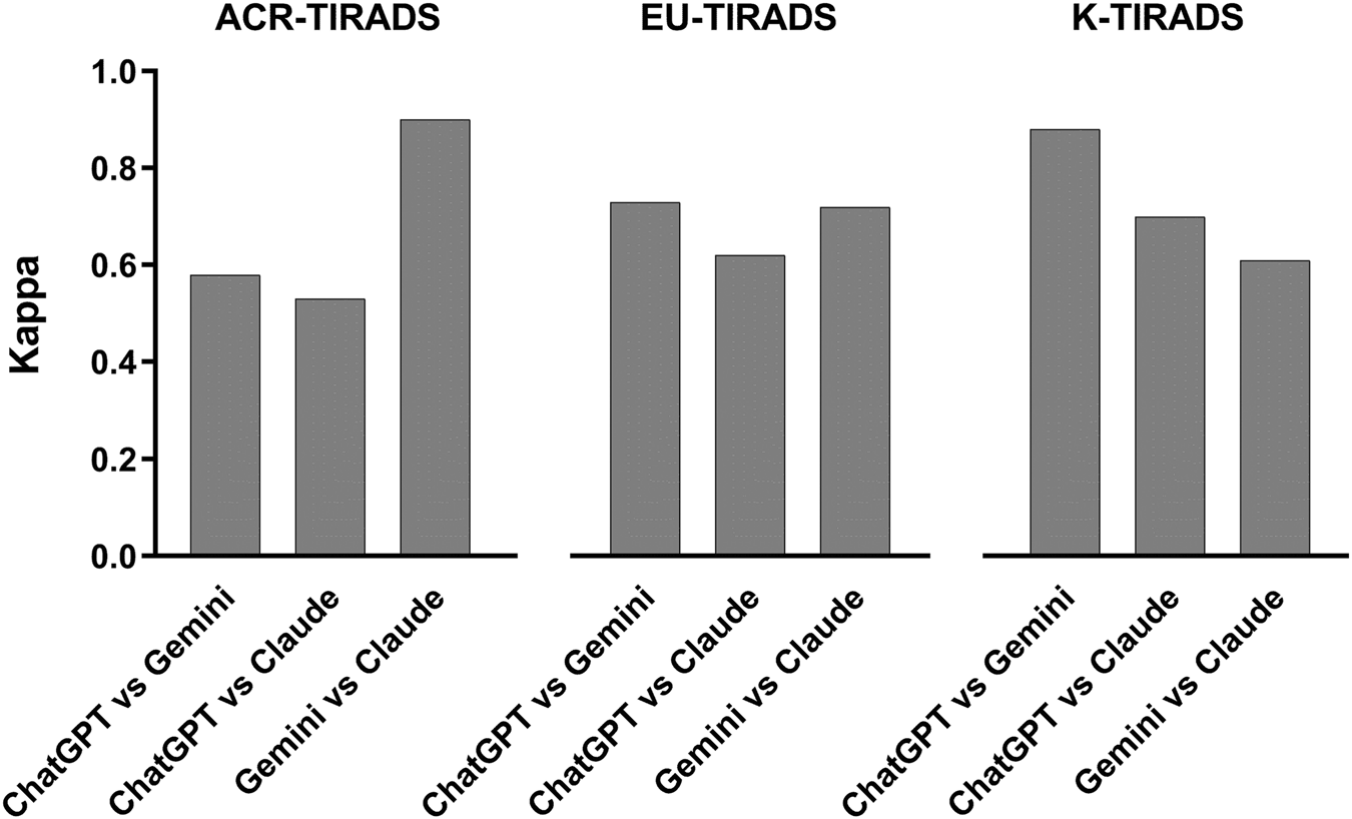
Interobserver agreement between AIs in assessing thyroid nodules across TIRADSs categories

According to the study design, 30 scenarios for each of the three TIRADSs were created. The assessment of the 90 cases according to the three AIs is illustrated in Fig. 1 and is described as follows. ACR-TIRADS: 100% of the cases were classified as category 2, 4, or 5 by ChatGPT, 90% were assessed as category 1, 2, 4, or 5 by Gemini, and 100% were classified as category 1, 2, or 5 by Claude. The IOA analysis gave a κ = 0.58 between ChatGPT and Gemini, 0.53 between ChatGPT and Claude, and 0.90 between Gemini and Claude. EU-TIRADS: 100% of the cases were classified as categories 2–5 by ChatGPT, 100% were classified as category 1, 2, 4, or 5 by Gemini, and 100% were classified as category 2, 3, or 4 according to Claude. The IOA analysis gave a κ = 0.73 between ChatGPT and Gemini, 0.62 between ChatGPT and Claude, and 0.72 between Gemini and Claude. K-TIRADS: 100% of the TNs were classified as category 3, 4, or 5 by ChatGPT and Gemini and as category 3 or 5 by Claude. The IOA analysis gave a κ = 0.88 between ChatGPT and Gemini, 0.70 between ChatGPT and Claude, and 0.61 between Gemini and Claude. The IOA findings are summarized in Fig. 2.

## Discussion

With the global diffusion of different TIRADS [12], standardization of the lexicon has become a hot topic in the field of thyroid US [5]. On the one hand, US operators must ask themselves if they actually adhere to what eminent societies have defined as the standardized terminologies to describe TNs. On the other hand, all users have to consider that AI has been rapidly diffused throughout medicine and that non-medical personnel and patients can also access online tools to better understand their clinical risks. In the thyroid field, although AI is gaining momentum, no strong results have been published about the performance of AIs in assessing the RoM of TNs on the basis of their US written description. Therefore, we conducted the present study to assess the IOA between AIs in their risk assessments according to the three major TIRADSs. These data are relevant when we consider AI as a potential tool to improve our clinical practice, and the data can also intrinsically help us to evaluate the capability of AI in understanding the US terminology.

The present study results can be summarized as follows. 1) Non-negligible heterogeneity is observed between AIs in the assessment of TNs according to the three TIRADSs. ChatGPT shows lower risk assessment with ACR-TIRADS, Claude divides the TNs into low- or high-risk classes with EU- and K-TIRADS, and Gemini assesses some cases as high-risk with ACR- and K-TIRADS but none with EU-TIRADS. 2) The IOA between AIs is generally moderate to good. 3) The value of κ tends to change according to the TIRADSs: the IOA between ChatGPT and Gemini is lowest with ACR-TIRADS, increases with EU-TIRADS, and is higher with K-TIRADS. A similar trend is observed for the IOA between ChatGPT and Claude, and an inverse trend is observed for the IOA between Gemini and Claude. This intrinsically means that the IOA can depend on the systems compared. To the best of our knowledge, this is the first data concerning the IOA between AIs in assessing the RoM of TNs according to the TIRADS categories.

In the field of medical US, AI-enhanced tools can guide human operators in real time, improving image acquisition, probe positioning, and reproducibility. From this point of view, AI is expected to transform healthcare, particularly for imaging and diagnostics. Although AI applications on medical images include the automated detection of lesions, accurate organ segmentation, and quantitative analysis of features, further advancements are warranted in the AI understanding of the written lexicon. All AI tools learn from data reported in online documents. To develop AI tools, researchers initially created algorithms that attempted to imitate the reasoning of humans. These algorithms are based on all types of written language that constantly change over time with human progress. The present results demonstrate that AI shows promise for understanding the terminology of TIRADSs, but important differences are observed between the tested AIs. The suboptimal IOA between the AIs we observed can be due to the differences in conceptualization between the TIRADSs. The three TIRADSs were conceived in the USA, Europe, and Asia, and had significantly different cultural contexts (i.e., health systems, costs, national programs, patient opportunities, hospital access, availability of medical procedures, and physician–patient communication) [7]. In addition, although the EU- and K-TIRADS are pattern-based systems, ACR-TIRADS is point based. Furthermore, ACR-TIRADS was basically conceived to reduce costs from FNACs and the literature has proven this assumption to be true [13, 14]. As we recently showed, these features can influence the different understandings of terminology by computer scientists even if they work in the medical field [15]. Consequently, the present findings should be of interest to computer/AI specialists and warrant broad discussion among this research community.

The present study had limitations that need to be discussed. The TIRADS scenarios were randomly selected, and their categories were differently represented in the 90 cases. However, this strategy was intended to avoid bias from predefined series. Second, the structures of the three TIRADSs and their US descriptors differ from each other. However, the various descriptors can sound quite similar to human operators.

In conclusion, our study found that there was non-negligible variability among the three AI tools tested for assessing the RoM of TNs across TIRADSs categories. These results have high relevance for future advancements in this field and should be of particular interest to the researchers involved in the ongoing international project to create the I-TIRADS. Clinicians and patients should be aware of these new findings.

## Data Availability

Database generated during and/or analyzed during the current study is available from the corresponding author upon reasonable request.
